# Supplementary material for: Investigation of skin microbiota reveals Mycobacterium ulcerans-Aspergillus sp. trans-kingdom communication
Source: Sci Rep. 2021 Feb 12;11:3777. doi: 10.1038/s41598-021-83236-7 (PMC7881091; doi:10.1038/s41598-021-83236-7)
Supplement: Supplementary file 1 — Supplementary Table 1. [file 41598_2021_83236_MOESM1_ESM.docx]

**Investigation of skin microbiota reveals *Mycobacterium ulcerans*-*Aspergillus* sp. trans-kingdom communication.**

Hammoudi N.^1,2^, Cassagne C^3^, Million M.^2^, Ranque S^3^, Kabore O.^2^,

Drancourt M.^2^, Zingue D. ^2,4^ Bouam A.^2*^

**Supplementary Table 1:** Primers used in this study for the PCR-based identification of fungi and bacteria.

|  | **Name** | **Sequences 5’-3’** |
| --- | --- | --- |
| Fungal sequences | ITS3 | GCATCGATGAAGAACGCAGC |
|  | ITS4 | TCCTCCGCTTATTGATATGC |
|  | Bt2a | GGTAACCAAATCGGTGCTGCTTTC |
|  | Bt2b | ACCCTCAGTGTAGTGACCCTTGGC |
|  | Al33F | GAYTTCATCAAGAACATGAT |
|  | Al33R | GACGTTGAADCCRACRTTGTC |
|  | Al34F | TTCATCAAGAACATGAT |
|  | Al34R | GCTATCATCACAATGGACGTTCTTGGAG |
|  | ITS1 | TCCGTAGGTGAACCTGCGG |
|  | ITS2 | GCTGCGTTCTTCATCGATGC |
| Bacterial sequences | FD1 | AGAGTTTGATCCTGGCTCAG |
|  | rP2 | ACGGCTACCTTGTTACGACTT |
|  | 536F | CAGCAGCCGCGGTAATAC |
|  | 536R | GTATTACCGCGGCTGCTG |
|  | 800F | ATTAGATACCCTGGTAG |
|  | 800R | CTACCAGGGTATCTAAT |
|  | 1050F | TGTCGTCAGCTCGTG |
|  | 1050R | CACGAGCTGACGACA |
