# Supplementary material for: Investigation of skin microbiota reveals Mycobacterium ulcerans-Aspergillus sp. trans-kingdom communication
Source: Sci Rep. 2021 Feb 12;11:3777. doi: 10.1038/s41598-021-83236-7 (PMC7881091; doi:10.1038/s41598-021-83236-7)
Supplement: Supplementary file 2 — Supplementary Table 2. [file 41598_2021_83236_MOESM2_ESM.docx]

**Investigation of skin microbiota reveals *Mycobacterium ulcerans*-*Aspergillus* sp. trans-kingdom communication.**

Hammoudi N.^1,2^, Cassagne C^3^, Million M.^2^, Ranque S^3^, Kabore O.^2^,

Drancourt M.^2^, Zingue D. ^2,4^ Bouam A.^2*^

**Supplementary Table 2:** Factor discriminant analysis (FDA) revealed five fungal and bacterial species that significantly discriminated *M. ulcerans*-PCR positive and negative samples.

| Variable | Lambda | F | DDL1 | DDL2 | p-value |
| --- | --- | --- | --- | --- | --- |
| *Propionimicrobium lymphophilum*-0 | 0,667 | 5,000 | 1 | 10 | 0,049 |
| *Brevundimonas diminuta*-0 | 0,500 | 10,000 | 1 | 10 | 0,010 |
| *Pantoea dispersa*-0 | 0,667 | 5,000 | 1 | 10 | 0,049 |
| *Penicillium rubens*-0 | 0,667 | 5,000 | 1 | 10 | 0,049 |
| *Aspergillus flavus*-0 | 0,286 | 25,000 | 1 | 10 | 0,001 |
