# Supplementary material for: Investigation of skin microbiota reveals Mycobacterium ulcerans-Aspergillus sp. trans-kingdom communication
Source: Sci Rep. 2021 Feb 12;11:3777. doi: 10.1038/s41598-021-83236-7 (PMC7881091; doi:10.1038/s41598-021-83236-7)
Supplement: Supplementary file 3 — Supplementary Table 3. [file 41598_2021_83236_MOESM3_ESM.docx]

**Investigation of skin microbiota reveals *Mycobacterium ulcerans*-*Aspergillus* sp. trans-kingdom communication.**

Hammoudi N.^1,2^, Cassagne C^3^, Million M.^2^, Ranque S^3^, Kabore O.^2^,

Drancourt M.^2^, Zingue D. ^2,4^ Bouam A.^2*^

**Supplementary Table 3:** accession numbers of bacteria and fungi sequences isolated in this study.

| SeqID1 | Bacillus drentensis | SUB8540265 | MW243007 |
| --- | --- | --- | --- |
| SeqID10 | Bacillus pumilus | SUB8540265 | MW243008 |
| SeqID11 | Bacillus subtilis | SUB8540265 | MW243009 |
| SeqID12 | Brevundimonas diminuta | SUB8540265 | MW243010 |
| SeqID13 | Cellulomonas iranensis | SUB8540265 | MW243011 |
| SeqID14 | Cellulomonas massiliensis | SUB8540265 | MW243012 |
| SeqID15 | Cellulomonas parahominis | SUB8540265 | MW243013 |
| SeqID16 | Cellulosimicrobium cellulans | SUB8540265 | MW243014 |
| SeqID17 | Collinsella massiliensis | SUB8540265 | MW243015 |
| SeqID18 | Curtobacterium citreum | SUB8540265 | MW243016 |
| SeqID19 | Enterococcus malodoratus | SUB8540265 | MW243017 |
| SeqID2 | Bacillus marasmi | SUB8540265 | MW243018 |
| SeqID20 | Franconibacter pulveris | SUB8540265 | MW243019 |
| SeqID22 | Lactobacillus plantarum | SUB8540265 | MW243020 |
| SeqID23 | Microbacterium arborescens | SUB8540265 | MW243021 |
| SeqID24 | Microbacterium hydrocarbonoxydans | SUB8540265 | MW243022 |
| SeqID25 | Microbacterium lacticum | SUB8540265 | MW243023 |
| SeqID26 | Microbacterium paludicola | SUB8540265 | MW243024 |
| SeqID27 | Microbacterium testaceum | SUB8540265 | MW243025 |
| SeqID28 | Micrococcus luteus | SUB8540265 | MW243026 |
| SeqID29 | Micrococcus yunnanensis | SUB8540265 | MW243027 |
| SeqID3 | Achromobacter spanius | SUB8540265 | MW243028 |
| SeqID30 | Ochrobactrum haemophilum | SUB8540265 | MW243029 |
| SeqID31 | Paenibacillus alvei | SUB8540265 | MW243030 |
| SeqID32 | Paenibacillus yonginensis | SUB8540265 | MW243031 |
| SeqID33 | Pantoea anthophila | SUB8540265 | MW243032 |
| SeqID34 | Pantoea stewartii | SUB8540265 | MW243033 |
| SeqID35 | Pantoea terrea | SUB8540265 | MW243034 |
| SeqID36 | Porphyromonas uenonis | SUB8540265 | MW243035 |
| SeqID37 | Propionibacterium freudenreichii | SUB8540265 | MW243036 |
| SeqID38 | Propionimicrobium lymphophilum | SUB8540265 | MW243037 |
| SeqID39 | Pseudomonas aeruginosa | SUB8540265 | MW243038 |
| SeqID4 | Acidovorax temperans | SUB8540265 | MW243039 |
| SeqID40 | Pseudomonas stutzeri | SUB8540265 | MW243040 |
| SeqID41 | Roseomonas gilardii | SUB8540265 | MW243041 |
| SeqID42 | Staphylococcus capitis | SUB8540265 | MW243042 |
| SeqID43 | Lelliottia nimipressuralis | SUB8540265 | MW243043 |
| SeqID44 | Lactobacillus reuteri | SUB8540265 | MW243044 |
| SeqID5 | Bacillus benzoevorans | SUB8540265 | MW243045 |
| SeqID6 | Bacillus marisflavi | SUB8540265 | MW243046 |
| SeqID7 | Bacillus megaterium | SUB8540265 | MW243047 |
| SeqID8 | Bacillus niacini | SUB8540265 | MW243048 |
| SeqID9 | Bacillus paralicheniformis | SUB8540265 | MW243049 |
| SeqID45 | Homoserinibacter gongjuensis | SUB8541717 | MW250249 |
| SeqID1 | dothideomycete | SUB8540314 | MW246088 |
| SeqID2 | Exophiala spinifera | SUB8540314 | MW246089 |
| SeqID3 | Gymnascella aurantiaca | SUB8540314 | MW246090 |
| SeqID4 | Ogataea thermomethanolica | SUB8540314 | MW246091 |
| SeqID5 | Purpureocillium lilacinum | SUB8540314 | MW246092 |
| SeqID6 | Rhizopus oryzae | SUB8540314 | MW246093 |
| SeqID7 | sordariomycete sp. | SUB8540314 | MW246094 |
| SeqID1 | Exophiala xenobiotica | BankIt2408451 | MW375131 |
| SeqID2 | Penicillium commune | BankIt2408456 | MW375132 |
| SeqID3 | Cryptococcus laurentii | BankIt2408462 | MW375133 |
